# The Unmeasured Burden of Febrile, Respiratory, and Diarrheal Illnesses Identified Through Active Household Surveillance in a Low Malaria Transmission Setting in Southern Zambia

**DOI:** 10.4269/ajtmh.21-1253

**Published:** 2022-06-15

**Authors:** Alexandra K. Mueller, Japhet Matoba, Jessica L. Schue, Harry Hamapumbu, Tamaki Kobayashi, Jennifer C. Stevenson, Philip E. Thuma, Amy Wesolowski, William J. Moss, for the Southern and Central African International Center of Excellence for Malaria Research

**Affiliations:** ^1^ Department of Pediatrics, Johns Hopkins School of Medicine, Baltimore, Maryland;; ^2^ Macha Research Trust, Choma District, Zambia;; ^3^ Department of International Health, Johns Hopkins Bloomberg School of Public Health, Baltimore, Maryland;; ^4^ Department of Epidemiology, Johns Hopkins Bloomberg School of Public Health, Baltimore, Maryland;; ^5^ W. Harry Feinstone Department of Molecular Microbiology and Immunology, Johns Hopkins Bloomberg School of Public Health, Baltimore, Maryland

## Abstract

Malaria incidence has declined in southern Zambia over recent decades, leading to efforts to achieve and sustain malaria elimination. Understanding the remaining disease burden is key to providing optimal health care. A longitudinal study conducted in a rural area of Choma District, Southern Province, Zambia, assessed the prevalence of and factors associated with symptoms of non-malarial illnesses and treatment-seeking behavior. We analyzed data collected monthly between October 2018 through September 2020 from 1,174 individuals from 189 households. No incident malaria cases were detected by rapid diagnostic tests among febrile participants. Mixed-effects logistic regression identified factors associated with cough, fever, diarrhea, and treatment-seeking. Incidence rates of cough (192 of 1,000 person-months), fever (87 of 1,000 person-months), and fever with cough (37 of 1,000 person-months) were highest among adults older than 65 years. Diarrhea incidence (37 of 1,000 person-months) was highest among children younger than 5 years. For every additional symptomatic household member, one’s odds of experiencing symptoms increased: cough by 47% (95% CI, 40–55), fever by 31% (95% CI, 23–40), diarrhea by 31% (95% CI, 17–46), and fever with cough by 112% (95% CI, 90–137), consistent with household clustering of illnesses. However, between 35% and 75% of participants did not seek treatment for their symptoms. Treatment-seeking was most common for children 5 to 9 years old experiencing diarrhea (adjusted odds ratio, 3.61; 95% CI, 1.42–9.18). As malaria prevalence reduces, respiratory and diarrheal infections persist, particularly among young children but, notably, also among adults older than 65 years. Increasing awareness of the disease burden and treatment-seeking behavior are important for guiding resource re-allocation as malaria prevalence declines in this region.

## INTRODUCTION

Malaria is a major source of morbidity and mortality, resulting in an estimated 627,000 deaths worldwide in 2020.^1^ The WHO African region is heavily impacted by malaria, with 96% of global deaths resulting from malaria in 2020 occurring in this region, and approximately 93% of malaria deaths occurring in just 32 countries in sub-Saharan Africa. Malaria is among the top 10 causes of death in Zambia; however, the burden of malaria in some endemic regions, including Southern Province, Zambia, has decreased significantly during the past two decades.^2^ The national prevalence of malaria in Zambia among children younger than 5 years decreased from 17% to 9% between 2015 and 2019, and Southern Province has one of the lowest malaria incidence rates in Zambia.^3^ As this region approaches malaria elimination, other causes of clinical illness become a higher priority. Diarrheal diseases and lower respiratory infections (LRIs) remain important causes of morbidity and mortality globally, particularly among young children. Although LRIs and diarrheal disease are prevalent globally, risk factors for poor disease outcomes, such as limited access to health care, inadequate nutrition, and poor sanitation, are associated with lower socioeconomic status, placing an inequitable risk for disease and severe outcomes on low-income countries.^4^ In sub-Saharan Africa, the mortality rate across all ages resulting from diarrhea is 61.8 deaths per 100,000, and the mortality rate for LRIs is 66.4 deaths per 100,000.^4^ Within Zambia, LRIs and diarrheal disease were both among the top five causes of death in 2018.^2^ Understanding risk factors for symptoms of LRIs and diarrheal disease can ultimately guide work to determine how the epidemiology of these diseases will be shaped after the decline of malaria, and how limited health resources should be re-allocated. It will also be important to investigate factors that motivate people to seek health care, as concern for malaria may have previously been one of the most important drivers.

This study used data collected as part of a longitudinal cohort study in Choma District, Southern Province, Zambia, in which households were surveyed monthly from October 2018 to September 2020. Although previous studies have investigated risk factors associated with signs and symptoms of illness using annual data from the cross-sectional Demographic and Health Survey, our study uses longitudinal data to look at seasonal trends over time.^5^^,^^6^ In addition, past studies have focused on the burden of respiratory infections using data from health centers.^7^ These passive surveillance data fail to capture individuals who do not seek treatment, which may lead to selection bias and an underestimate of the disease burden.^8^ Our study, however, used active surveillance of households to collect data, with frequent visits independent of individuals seeking care. In addition to providing a more accurate estimate of the prevalence and incidence of illness, a better understanding of individuals who do not seek care can also be gained through active surveillance. Therefore, through the longitudinal nature of our study, we can estimate the incidence rate of symptoms of disease as well as the burden of disease among people who do not seek treatment.

## METHODS

### Ethics.

Adult participants provided signed informed consent, and parents or guardians of children provided parental permission. The study was approved by the Institutional Review Board of the Johns Hopkins Bloomberg School of Public Health and the Ethics Review Committee of the Tropical Diseases Research Center.

### Study population.

A prospective longitudinal cohort study, the Antoomwe cohort study, surveyed households in the rural catchment area of Mapanza Rural Health Center in Choma District, Southern Province, Zambia (Supplemental Figure S1). In Zambia, most residents live with extended families comprising one or more households.^9^ This study’s sampling frame was chosen for its historically higher level of malaria incidence. Encompassing approximately 2 km^2^, the sampling frame’s perimeter was defined by natural borders such as roads and trails. Satellite imagery and maps were used to locate households within the sampling frame. After obtaining consent from the head of each household, a census was used to enumerate all households in the catchment area. Individual surveys were administered to household members in the sampling frame once a month for 2 years between October 2018 through September 2020, with a pause in data collection between April and June 2020 because of COVID-19 restrictions. Thus, the maximum number of study visits per household was 22. These monthly surveys included questions on demographic characteristics, travel, symptoms experienced in the past day and during the previous 2 weeks, known malaria risk factors and prevention efforts, and malaria testing and results. The household census data were combined with the individual survey data to provide detailed, longitudinal information about the households and individual participants.

The cohort was designed to describe malaria transmission and risk in Southern Province, Zambia, a low transmission setting. During the study period, all participants were tested for the presence of *Plasmodium falciparum* using quantitative polymerase chain reaction. To test for incident clinical malaria, any individual with a fever was tested for infection using a rapid diagnostic test (RDT). There were no incident clinical malaria cases detected by RDT among febrile cohort participants. Some individuals, however, had sub-patent *P. falciparum* infection detected by quantitative polymerase chain reaction.^9^ Therefore, the study was able to investigate symptoms unrelated to incident RDT-positive malaria.

### Inclusion and exclusion criteria.

**Figure 1.**
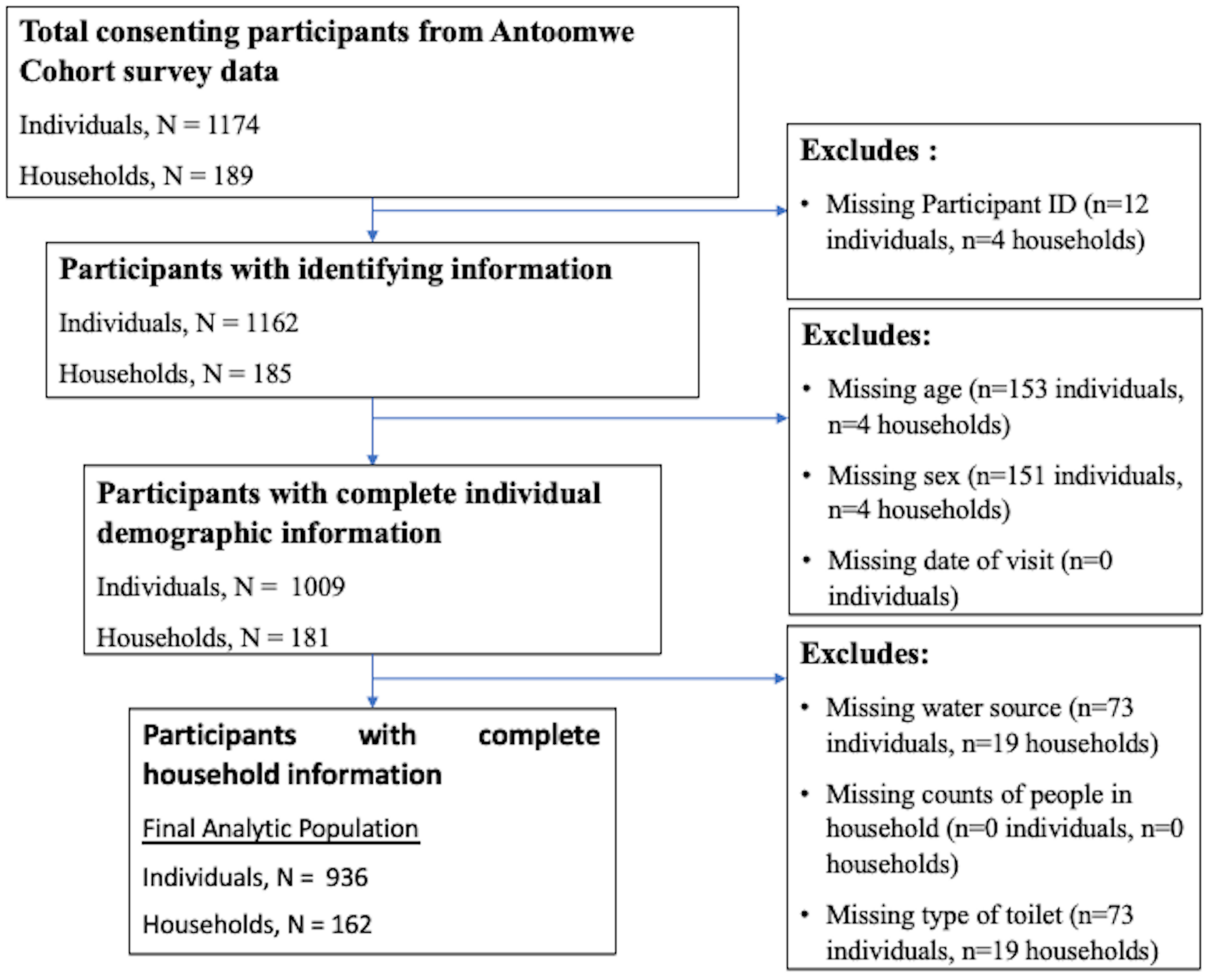
Study population inclusion and exclusion criteria.

Residents and visitors living in households within the sampling frame were eligible to participate in the study. All household members 3 months of age or older were eligible to participate in the study. Surveys were administered to participants each month. Survey responses with a household ID that matched a household ID in the census were included in the final analyses. Responses missing a participant ID, visit date, age, gender, and household characteristics were excluded from the analytic data set (Figure 1).

### Measures.

To assess symptoms experienced by individuals, participants were asked whether they had experienced a fever, cough, or diarrhea in the past day and prior 2 weeks. The caregiver of participants too young to respond answered the survey for children. To approximate LRI, individuals were classified as having “fever with cough” if they concurrently reported fever and cough.^10^ Participants were considered symptomatic if they experienced one of the symptoms within the past day, and every occurrence of a symptom in the past day was defined as an incident symptomatic event. Each visit date was categorized into one of Zambia’s three major seasons; warm and wet, December through April; cool and dry, May through August; and hot and dry, September through November.

At the first census visit, each household self-reported the main source of drinking water (piped water; borehole, bush pump, protected well; open, unprotected well; surface water, stream, pond, or other) and the type of toilet typically used in the home (owned flush toilet, owned pit latrine, shared pit latrine, bush/open defecation, or other). The number of people in each household was measured at the first census, along with the gender and education level of the head of the household. The number of sick people in each household was measured by totaling the symptomatic individuals in a house at each visit. In addition, the distance between the health center and each home was calculated using Euclidean distance between each home’s Universal Transverse Mercator coordinates and the Mapanza Rural Health Center Universal Transverse Mercator coordinates.

### Statistical analysis.

During the exploratory data analysis, the distribution of all variables of interest was evaluated to understand general characteristics of the cohort (Table 1) and the level of completeness of the data. The variables of interest were selected based on risk factors for diarrhea, fever, and cough identified in previous studies conducted in sub-Saharan Africa.^11^^–^^14^ Age, gender, season, and self-report of symptoms at any previous visit were included as individual risk factors. Age was measured at baseline and was held constant for the analyses. Age was stratified into four categories: 0 to 4 years, 5 to 9 years, 10 to 14 years, and 15 years and older. Because previous studies showed that diarrhea and pneumonia are associated significantly with children’s age, the age groups were analyzed for those younger than 15 years of age with the greatest granularity.^11^ Because of the potential heterogeneity in risk factors among adults, a fifth age group, 65 years and older, was created to explore the incidence rate and treatment-seeking behavior in these individuals. This age group had a small sample size and was not included in the mixed-effects models. Household variables of interest included water source, toilet type, gender and education level of the head of the household, and number of people per home. These characteristics were measured at baseline and were held constant throughout the study. Counts of sick individuals per household were included to estimate the impact of living with symptomatic household members.

**Table 1:** Summary of characteristics among participants in the final analytic study population between September 2018 and October 2020 (*N* = 936)

Individual characteristics at baseline	*n* (%)*
Age, years	
0–4	127 (13.6)
5–9	142 (15.2)
10–14	151 (16.1)
15–64	484 (51.7)
≥ 65	32 (3.4)
Age, median (IQR)	16.5 (20.3)
Gender	
Male	413 (44.1)
Female	523 (55.9)
Report of symptom at least once during follow-up	
Fever in past day	309 (33.0)
Cough in past day	483 (51.6)
Diarrhea in past day	156 (16.7)
Fever with cough in past day	142 (15.2)
Report of symptom at least 3× during follow-up	
Fever in past day	47 (5.0)
Cough in past day	158 (16.9)
Diarrhea in past day	12 (1.3)
Fever and cough in past day	10 (1.1)
Household characteristics at baseline (*n* = 162)	
Water source	
Bore hole, bush pump, or protected well	112 (69.1)
Open or unprotected well	9 (5.6)
Piped water	41 (25.3)
Toilet type	
Owned pit latrine	122 (75.3)
Shared pit latrine	40 (24.03)
Gender, head of household	
Male	80 (54.1)
Female	68 (45.9)
Years of education, head of household	
0–1	5 (3.2)
2–7	48 (31.2)
8–9	12 (7.8)
≥ 10	89 (47.8)
No. of children in the house, median (IQR)	2 (4)
No. of people in the house, median (IQR)	5 (4)

IQR = interquartile range.

* Values presented as number (percentage) unless noted otherwise.

Missing data were characterized and considered missing at random (Supplemental Table S1). A complete case analysis method was used. Any observations missing data on household ID, participant ID, age, gender, visit date, water source, type of toilet, or the number of persons in the household were excluded. Because of a high percentage of data missing head-of-household information, missing data were allowed for this variable, but head-of-household information was not included in the final models.

The frequency and recurrence of symptoms were investigated to determine the most common symptoms and how often individuals experienced fever, cough, or diarrhea at consecutive visits. Histograms were constructed to depict the distribution of reports of each symptom. Furthermore, the seasonal pattern of each symptom was explored by plotting the incidence rate of each symptom against each season and month, which was compared with the seasonal and monthly reports of patients with diarrhea and pneumonia seeking care as reported by Mapanza Rural Health Center.

The odds of experiencing fever, cough, and diarrhea in this cohort were investigated using mixed-effects univariable and multivariable logistic regression, with random effects included for the individual and the household. Random effects were included to allow for within-person and within-household random variability across visits, and to account for the repeated observations at the person and household levels. Age and gender were included in the multivariable models, as well as variables that showed significance at an alpha level of 0.05 in the univariable logistic regression. Age-adjusted mixed-effects logistic regression was used to explore the odds of seeking treatment for fever, cough, and diarrhea, with a random effect included for the household. An unstructured covariance matrix was used in the mixed-effects models to allow for each variance and covariance to be estimated uniquely. The percentage of variance resulting from clustering at individual and household levels was assessed through intraclass coefficient (ICC) values. Random effects for individual and household levels were included in models with outcomes related to experiencing symptoms, because ICC values were more than 0.1 for both the individual and household levels. The ICC values were less for the outcomes related to treatment-seeking. Therefore, we only used a random effect for the household level. Within-person and within-household correlations were low, with an ICC less than 0.1 for both the household and individual IDs in each model after including the random effects. Models with the lowest Akaike information criterion were ultimately chosen for the final analysis. Models were performed using R version 4.0.1 (R Foundation for Statistical Computing, Vienna, Austria).^15^

## RESULTS

The cohort included 13,732 observations, of which 11,970 observations had household IDs that were matched between the census and participant surveys, and had complete data on participant ID, age at baseline, gender, the season of the visit, water source, type of toilet, number of children, and total people in the household (Figure 1). This included 936 individual participants residing in 162 houses. Visits spanned from October 2018 through September 2020, and the median number of visits attended per participant was 15 visits (interquartile range [IQR], the difference between the 75th percentile and the 25th percentile of the data: 10). On average, there were 73.6 weeks between participants’ first and last study visits. The characteristics of the analytic population, which excluded observations missing data on household ID, participant ID, age, gender, visit date, water source, type of toilet, or the number of persons in the household, was compared with the characteristics of observations excluded from the study. The median age in both cohorts was 16.5 years (observations excluded IQR 17.0 and analytic population 20.3), and approximately 40% of both populations were male. Student’s *t*-test and χ^2^ tests were used to compare age; gender; reports of fever, cough, or diarrhea; and median number of visits attended between observations excluded from the data set and the analytic population (Supplemental Table S1). The excluded observations had significantly fewer reports of lower cough, fever, and diarrhea per person and a significantly fewer median number of visits per person (*n *= 3; IQR, 4 versus *n *= 15; IQR, 4.5; *P* < 0.001) compared with the analytic population. Neither age nor gender were significantly different between observations excluded and those in the analytic population. Those excluded from the analysis were assumed to be represented by those included in the analysis.

Of the 936 individuals, 13.6% were younger than 5 years, and the median age was 16.5 years (IQR, 20.3; Table 1). The 65-years-and-older group comprised only 3.4% of the analytic population. Given this age group’s small size, it was not included in the mixed-effects models. At baseline, most households’ main water source was a bore hole, bush pump, or protected well (69.1%), and 75% of households owned a pit latrine. The median number of children younger than 16 years in each household was two (IQR, 4), and the median number of people in the household was five (IQR, 4).

Across all study visits throughout follow-up, 33.0% of all individuals reported fever in the past day, 51.6% reported cough in the past day, 16.7% reported diarrhea in the past day, and 15.2% reported fever with cough in the past day (Table 1). Some individuals reported having symptoms at multiple study visits. For example, 16.9% reported a cough in the past day at three or more study visits.

The overall incidence rate for cough was highest at 90.6 episodes of cough per 1,000 person-months followed by fever (39.4 episodes per 1,000 person-months) and diarrhea (17.1 episodes per 1,000 person-months; Table 2). The incidence rate of fever with cough was 14.7 episodes per 1,000 person-months. Despite an overall decrease in the incidence rate as age increased, when the category of those older than 65 years was included, incidence rates for both cough and fever among individuals 65 years and older were greater than those younger than 5 years.

**Table 2:** Incidence rates for symptoms of illness stratified by age, season, and gender, Choma District, Southern Province Zambia, 2018–2020 (*N* = 936)

Characteristic	Incidence rate of fever, (fever/person-month) ×1,000	Incidence rate of cough (cough/person-month) ×1,000	Incidence rate of diarrhea, (diarrhea/person-month) ×1,000	Incidence rate of fever with cough, (fever and cough/person-month) ×1,000)
Overall	39.4	90.6	17.1	14.7
Age, years				
0–4	66.8	138.5	36.5	34.0
5–9	35.8	94.6	10.2	13.9
10–14	21.9	80.8	15.5	6.5
15–64	34.7	70.25	14.15	10.44
≥ 65	86.88	192.24	22.18	37.0
Season				
Warm and wet	39.8	85.3	16.7	14.3
Hot and dry	43.6	101.4	21.0	17.0
Cool and dry	35.4	88.1	14.4	13.3
Gender				
Male	37.3	90.1	17.5	11.3
Female	41.1	90.9	16.8	17.3

A seasonal pattern in incidence rates was observed for all symptoms. Among all participants, the adjusted odds of experiencing symptoms increased during the hot and dry season. The individual and household specific adjusted odds of cough during the hot and dry season was 1.21 (95% CI, 1.02–1.44) times the odds of cough during the cool and dry season (Table 3). The individual and household specific adjusted odds ratio (aOR) of diarrhea comparing the hot and dry season to the cool and dry season was 1.53 (95% CI, 1.06–2.21). The aOR for fever alone and fever with cough were elevated for the hot and dry season but were not statistically significantly higher. In analyses of children younger than 5 years (*n* = 127) and children younger than 15 years (*n* = 429), this seasonal trend was not observed, but the sub-cohort of children younger than 5 years may have been underpowered to detect differences.

**Table 3:** Adjusted logistic regression estimates of experiencing symptoms among cohort members, Choma District, Southern Province, Zambia, 2018 to 2020 (*N* = 936)

Variable*	Adjusted OR of fever (95% CI)	Adjusted OR of cough (95% CI)	Adjusted OR of diarrhea (95% CI)	Adjusted OR of fever with cough (95% CI)
Age, years				
0–4	Ref	Ref	Ref	Ref
5–9	0.55 (0.36–0.83)†	0.67 (0.48–0.94)†	0.26 (0.14–0.50)†	0.36 (0.19–0.70)†
10–14	0.31 (0.20–0.49)†	0.59 (0.42–0.82)†	0.43 (0.24–0.76)†	0.15 (0.07–0.33)†
≥ 15	0.64 (0.46–0.89)†	0.55 (0.42–0.72)	0.45 (0.29–0.70)†	0.32 (0.19–0.54)†
Gender				
Male	Ref	Ref	Ref	Ref
Female	1.05 (0.819–1.34)	0.99 (0.82–1.20)	0.93 (0.65–1.32)	1.52 (0.99–2.33)
Season				
Cool and dry	Ref	Ref	Ref	Ref
Hot and dry	1.25 (0.97–1.62)	1.21 (1.01–1.43)†	1.53 (1.06–2.21)†	1.48 (0.96–2.29)
Warm and wet	1.22 (0.96–1.56)	0.98 (0.83–1.15)	1.25 (0.870–1.78)	1.33 (0.87–2.02)
Diarrhea at current visit	2.93 (1.84–4.65)†	1.58 (1.05–2.35)†	NA	2.58 (1.32–5.04)†
Diarrhea at previous visit	1.41 (0.78–2.57)	1.40 (0.90–2.17)	0.96 (0.470–1.96)	1.90 (0.84–4.34)
Cough at current visit	5.14 (4.08–6.48)†	NA	1.97 (1.35–2.88)†	NA
Cough at previous visit	1.16 (0.86–1.58)	1.30 (1.05–1.62)†	1.76 (1.18–2.63)†	1.64 (1.02–2.66)
Fever at current visit	NA	5.15 (4.05–6.54)†	NA	NA
Fever at previous visit	NA	1.27 (0.93–1.73)	NA	1.49 (0.82–2.68)
No. of people sick in the household	1.31 (1.23–1.40)†	1.47 (1.40–1.55)†	1.31 (1.17–1.46)†	2.12 (1.90–2.37)†
No. of people with diarrhea in the household	NA	NA	1.03 (0.67–1.6)	NA

NA = not applicable; OR = odds ratio; Ref = reference value.

* Each covariate is adjusted for all other covariates included for the symptom. Covariates with NA values were not included in the model for that symptom.

† Significance level of *P* < 0.05

Seasonal trends by year were also investigated. Incidence rates of all symptoms were the greatest during the hot and dry season of 2018. During the hot and dry season of 2018, cough had the greatest incidence rate at 154 per 1,000 person-months, followed by fever at 82 per 1,000 person-month.

### Individual and household factors associated with signs and symptoms.

Age was associated significantly with the odds of each symptom (Table 3). Comparing older individuals to those younger than 5 years, the odds of experiencing an episode of fever, diarrhea, or fever with cough decreased significantly. For example, the individual and household-specific aORs of fever comparing children 5 to 9 years old, children 10 to 14 years, and adults 15 years and older to children younger than 5 years were 0.55 (95% CI, 0.36– 0.83), 0.31 (95% CI, 0.20–0.49), and 0.64 (95% CI, 0.46–0.89), respectively. This trend was similar for the cough, diarrhea, and fever with cough models (Table 3). There was no significant association observed between the participants’ gender and their odds of experiencing any of the symptoms.

The probability of experiencing each symptom was dependent on symptoms experienced at a prior visit. The adjusted odds of experiencing cough among those who had a cough at their prior visit were 30% greater compared with those without a cough at their last visit (Table 3). Those who experienced a cough at their previous visit had an aOR of 1.76 (95% CI, 1.18–2.63) of having diarrhea at the current visit compared with those without prior cough, adjusting for age, gender, season, diarrhea at the past visit, cough at the current visit, and the number of people sick in the house. No significant increase in the odds of fever based on previous visit symptoms was observed.

Household characteristics including water source, toilet type, number of children in the household, and number of people in the household were not associated significantly with an individual’s symptoms (Supplemental Table 2). However, individuals living in households with other persons experiencing any symptoms were significantly more likely to have a fever, cough, diarrhea, or fever with cough. For each one-person increase in the number of symptomatic people in the household, the adjusted odds of having a fever increased by 31% (aOR, 1.31; 95% CI, 1.23–1.40), the odds of having a cough increased by 47% (aOR, 1.47; 95% CI, 1.40–1.55), the odds of diarrhea increased by 31% (aOR, 1.31; 95% CI, 1.17–1.46), and the odds of fever with cough increased by 112% (aOR, 2.12; 95% CI, 1.90–2.37; Table 3).

Many participants experienced multiple symptoms concurrently. The aOR of cough comparing those who had fever at the same visit to those who did not was 5.15 (95% CI, 4.05–6.54). The aOR of cough comparing those who had diarrhea at the same visit to those who did not was 1.58 (95% CI, 1.05–2.35). Furthermore, the adjusted odds of diarrhea was 1.97 (95% CI, 1.35–2.88) times greater among people who also had a cough at the same visit. Experiencing diarrhea at the same visit increased the odds of having fever with cough by 158% (aOR, 2.58; 95% CI, 1.32–5.04). The odds of having a fever were also increased among those who experienced a cough concomitantly (aOR, 5.14; 95% CI, 4.08–6.48) or diarrhea (aOR, 2.93; 95% CI, 1.84–4.65; Table 3).

### Individual and household factors associated with seeking treatment.

**Figure 2.**
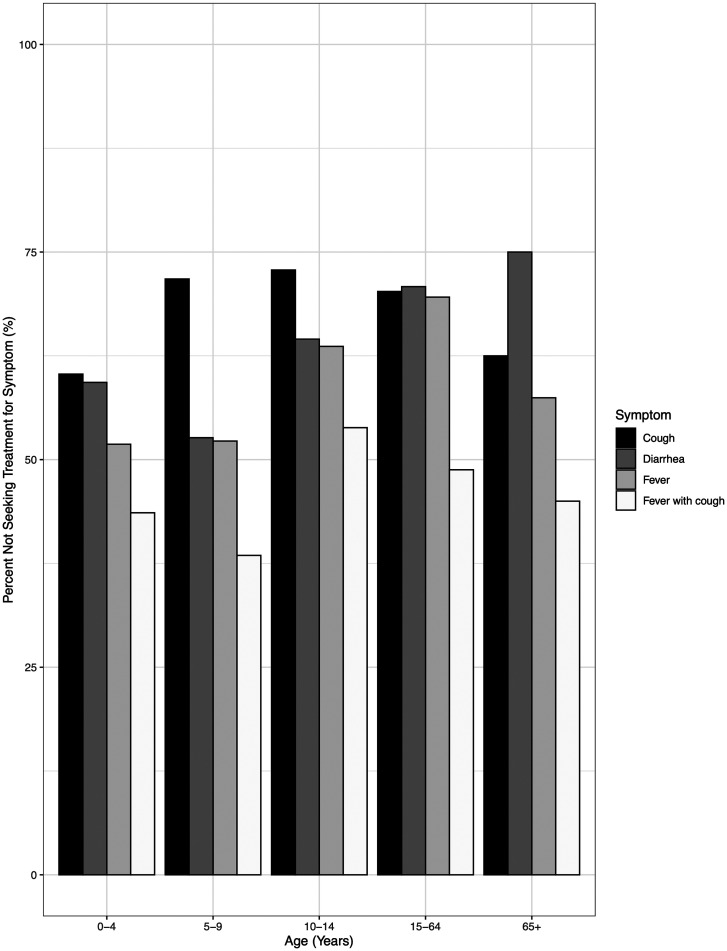
Proportion of individuals within each age category who did not seek treatment for their symptoms.

The odds of seeking treatment for each symptom based on individual and household characteristics were investigated. Notably, the individual and household-specific adjusted odds of seeking treatment for fever were significantly less among those 15 years and older compared with those younger than 5 years (aOR, 0.44; 95% CI, 0.257–0.753). Although the odds of seeking treatment for fever were less among those ages 5 to 9 and 10 to 14 years of age compared with those younger than 5 years, this reduction was not significant. The odds of seeking treatment for a cough among those 5 years and older were significantly less compared with those younger than 5 years. Furthermore, the odds of seeking treatment for diarrhea showed no significant difference across age groups. There was no significant difference in the odds of seeking treatment for fever with cough by age. Adults 65 years and older experiencing diarrhea had the highest percentage of episodes with no treatment sought; no treatment was sought for 75% of diarrheal episodes in this age group (Figure 2). Children between the ages of 5 and 9 years experiencing fever with cough comprised the greatest percentage of those who sought treatment (61.5%). No differences were observed in the probability of seeking treatment between males and females. In addition, treatment-seeking behavior showed no significant association with season.

Interestingly, the distance between the household and Mapanza Rural Health Center was not associated with the odds of seeking treatment for any symptom (fever OR, 0.793, 95% CI, 0.525–1.20; cough OR, 1.22, 95% CI, 0.909–1.65; diarrhea OR, 0.656, 95% CI, 0.359–1.20), although all households were in the health center catchment area. The median distance between each house and the health center was 6 km (IQR, 0.93 km; 5.3–8.4 km).

The number of symptomatic individuals in the household had an important impact on the odds of seeking treatment for each symptom (Table 4). The odds of seeking treatment for a fever increased as the number of symptomatic household residents increased. Specifically, the age-adjusted odds of seeking treatment for a fever increased by 28% (aOR, 1.28; 95% CI, 1.12–1.47) for each additional household member of any age experiencing any of the three symptoms. However, the odds of seeking treatment for any of the symptoms was not associated significantly with the number of symptomatic 0- to 4-year-olds in the household. For each additional symptomatic 5- to 9-year-old in the house, the age-adjusted odds of seeking treatment for a fever increased by 50% (aOR, 1.50; 95% CI, 1.09–2.05). But the odds of seeking treatment for any symptom was not associated significantly with the number of symptomatic 10- to 14-year-olds in the household. Each increase in the number of symptomatic adults in the household increased the age-adjusted odds of seeking treatment for fever by 49% (aOR, 1.49; 95% CI, 1.17–1.90). These trends were consistent for seeking treatment for cough and diarrhea.

**Table 4:** Mixed-effects multivariate logistic regression estimates of seeking treatment among cohort members, Choma District, Southern Province, Zambia, 2018 to 2020 (*N* = 936)

Variable*	Adjusted OR of seeking treatment for fever (95% CI)	Adjusted OR of seeking treatment for cough (95% CI)	Adjusted OR of seeking treatment for diarrhea (95% CI)	Adjusted OR of seeking treatment for fever with cough (95% CI)
Total no. of people sick in the house	1.28 (1.12–1.47)†	1.16 (1.07–1.26)†	1.17 (0.96–1.43)	0.96 (0.81–1.14)
No. of sick 0- to 4-year-old children	1.34 (0.99–1.81)	1.16 (0.96–1.41)	1.17 (0.73–1.85)	0.81 (0.54–1.21)
No. of sick 5- to 9-year-old children	1.50 (1.09–2.05)†	1.20 (0.99–1.45)	3.61 (1.42–9.18)†	1.09 (0.75–1.60)
No. of sick 10- to 14-year-old children	1.53 (0.99–2.35)	1.15 (0.87–1.53)	1.06 (0.61–1.85)	0.91 (0.44–1.88)
No. of sick adults	1.49 (1.17–1.90)†	1.37 (1.17–1.60)†	1.03 (0.74–1.45)	0.913 (0.61–1.36)

OR = odds ratio.

* Each covariate is adjusted for age.

† Significance level of *P* < 0.05.

## DISCUSSION

In communities where malaria transmission has declined substantially, characterizing symptoms of illness is important to help transition from malaria control to provision of health care that best meets the needs of the community. This longitudinal study, conducted in Southern Province, Zambia, is unique in providing longer follow-up time and more frequent study visits that capture more nuanced temporal patterns of symptoms of illness. In addition, through active household surveillance, the burden of disease among people who do not present to health clinics was captured. In this study of 936 individuals residing in 162 households, cough was the most frequently experienced symptom, followed by fever, with diarrhea being the least common. Although there was heterogeneity in trends for fever and cough from year to year, the peak incidence rates for fever, cough, and diarrhea were during the hot and dry season of 2018. Although our study is unable to draw diagnostic conclusions, previous studies found that influenza B peaks during the hot and dry season in this region, particularly among children younger than 5 years.^7^ Consistent with previous findings regarding demographic characteristics of respiratory infections, fever and cough were most prevalent among children younger than 5 years of age.^7^^,^^16^^,^^17^ Upon disaggregating the oldest age group, those 65 years and older had the highest incidence rates of fever and cough, and second highest incidence rate of diarrhea. This finding of high risk for cough among older adults is consistent with data reported by the WHO global health estimates.^18^ In 2016, the proportion of deaths caused by respiratory infections was greatest in children 0 to 4 years old, followed by adults 70 years and older.^19^ The Global Burden of Diseases, Injuries, and Risk Factors Study 2016, which is a large study encompassing 195 countries, including Zambia, that models the burden of disease for many illnesses including diarrhea and LRIs, found that although the global burden of LRIs was decreasing for children younger than 5 years, the burden of disease increased for adults older than 70 years.^17^ The greatest burden of LRIs among older adults was observed in countries ranked lower on the sociodemographic index.^17^ High incidence rates of LRIs and diarrhea combined with low levels of treatment-seeking among older adults is important to consider as communities age in Zambia and across sub-Saharan Africa.

Many individuals experienced multiple symptoms concurrently. Fever and cough were both more likely to occur if the participant also had diarrhea, and diarrhea was more likely to occur if the participant also had a cough. A study of Ethiopian children younger than 5 years reported significant co-occurrence of fever with cough and fever with diarrhea, but no association between cough and diarrhea.^20^ There is growing evidence that acute respiratory infections coincide with diarrhea.^21^^,^^22^ One study among Indian and Nepali children found that diarrhea and LRIs occurred together significantly more than would be expected by chance alone.^21^ In our analysis, the odds of diarrhea increased significantly for those with a previous report of a cough. The findings indicate cough may precede diarrhea during the course of illness.

Individuals residing in homes with more people experiencing fever, cough, and/or diarrhea increased the odds of having each symptom. Although this analysis is not able to draw conclusions about transmission pathways, the results are indicative of household clustering and possible within-household transmission. Infectious diarrhea has been shown to be transmitted within households,^23^ and household transmission has also frequently been demonstrated for illnesses associated with cough and fever.^24^^,^^25^ Our findings suggest it may be particularly salient to test and provide care to entire households when one person in a household becomes ill, similar to what is done for malaria in reactive test-and-treat strategies.^26^

Treatment-seeking behavior was also investigated. Participants often did not seek care for fever, cough, or diarrhea, perhaps because the illness was mild. In any one age group, more than half of individuals reporting fever, cough, or diarrhea did not seek care. People were more likely to seek care if they had fever and cough. Other factors associated with treatment-seeking were younger age and greater number of symptomatic household members. Previously, maternal education, distance to health clinics, financial barriers, gender of the head of the household, and social norms have all been cited as influencing treatment-seeking behavior.^27^ Similar to our study, an analysis of treatment-seeking for diarrheal illnesses in Gaza found that seeking treatment for diarrhea was more common if the child was younger than 5 years of age.^28^ Few, if any, studies have investigated the association between the number of sick individuals in the household and treatment-seeking. It is evident from this analysis that greater numbers of sick household members increased the odds of experiencing each symptom and of seeking treatment for each symptom. This could have important implications for better understanding the factors driving individuals to seek treatment.

Because of the large number and frequency of visits included in this cohort study, missing data were a challenge. If participants with missing data were significantly different than those with complete data, the results of this analysis may not be representative of the target population, resulting in emigration selection bias. In assessing missing data, those with incomplete data were more likely to have fewer visits attended and had a lower average number of cough episodes. One possible explanation for this is that healthier people were more likely to travel, leading to missed visits. An available data analysis or multiple imputation are both alternative methods of analysis that could have been used to handle the missing data. Because the survey measured signs and symptoms of illness, we were unable to draw conclusions about the actual associated diseases present in the community; however, future studies using diagnostic testing would be able to investigate this further. We assumed people stayed in the same household and resided with the same people throughout the study. Data collected from a second census conducted in September 2019 showed that many individuals moved into and out of households throughout the study. This increased our uncertainty of who was living in the household at each study visit. Finally, this study may also be limited in its external validity. This region of Zambia is rural and has a low incidence of malaria. The results of this study may not apply to other regions in Zambia or other settings in sub-Saharan Africa.

## CONCLUSION

Overall, this study helps characterize the burden of fever, cough, and diarrhea in a region with declining malaria transmission. Our results provide important insights into the unmeasured burden of fever, cough, and diarrhea, because it was able to capture episodes of illness not seen at health-care facilities. Cough had the highest incidence rate of all the symptoms. Children younger than 5 years and adults older than 65 years were at greatest risk of symptomatic illness. In addition, many symptoms occurred concurrently. Although the study is unable to draw conclusions on transmission chains, the odds of each symptom were increased when other sick people were present in the same household. Importantly, treatment-seeking also increased when other people in the same household were ill. Because individuals living with symptomatic household residents had a greater likelihood of becoming ill themselves, we recommend that integrated community case management (ICCM) advise community health workers to evaluate family members of ill residents for greater impact and efficiency. The analysis also helps us understand the influences associated with seeking treatment, particularly concurrent illness in other household residents. Further research into factors such as maternal education, nutrition, and the etiology of these symptoms may help to expand our understanding of the burden of disease in areas approaching malaria elimination.

## Supplemental Material


Supplemental materials
