# Supplementary material for: The Unmeasured Burden of Febrile, Respiratory, and Diarrheal Illnesses Identified Through Active Household Surveillance in a Low Malaria Transmission Setting in Southern Zambia
Source: Am J Trop Med Hyg. 2022 Jun 15;106(6):1791–9. doi: 10.4269/ajtmh.21-1253 (PMC9209922; doi:10.4269/ajtmh.21-1253)
Supplement: Supplementary file 1 [file tpmd211253.SD1.pdf]

**Supplemental Table S1.** Comparison of characteristics between the observations excluded from the analytic population and the analytic population.

| Characteristic | Observations excluded from analytic population (N=226) | Analytic population (N=936) | P-value (2 sample t-test) |
|---|---|---|---|
| | Median (IQR)* | Median (IQR)* | |
| **Average Age** | 17.0 (12.5) | 16.5 (20.25) | 0.07 |
| | n (%) | n (%) | |
| **Sex** | | | 0.25 |
| **Male** | 28 (37.3%) | 413 (44.1%) | |
| **Female** | 47 (62.7%) | 523 (55.9%) | |
| **Average total cough reports** | 0.30 | 1.19 | <0.001 |
| **Average total fever reports** | 0.17 | 0.54 | <0.001 |
| **Average total diarrhea reports** | 0.08 | 0.23 | <0.001 |
| **Total Cough>3** | 0 (0.0%) | 85 (9.1%) | <0.001 |
| **Total Fever>3** | 1 (0.4%) | 19 (2.0%) | 0.01 |
| **Total Diarrhea>3** | 0 (0.00%) | 3 (0.32%) | 0.08 |
| **Median number of visits attended** | 3.00 (4) | 15 (4.5) | <0.001 |

* IQR (interquartile range) is defined as the difference between the values, or spread, of the $75^{th}$ and $25^{th}$ percentiles of data.

**Supplemental Table S2.** Univariable mixed effects logistic regression estimates of impact of household level factors on odds of symptoms

| Variable | Odds Ratio of Fever (95% CI) | Odds Ratio of Cough (95% CI) | Odds Ratio of Diarrhea (95% CI) | Odds Ratio of Fever with cough (95% CI) |
|---|---|---|---|---|
| **Water source** | | | | |
| **Bore hole, bush pump, or protected well** | REF | REF | REF | REF |
| **Open or unprotected well** | 0.99 (0.52, 1.90) | 1.39 (0.78, 2.47) | 1.60 (0.59, 4.32) | 1.02 (0.40, 2.60) |
| **Piped water** | 1.11 (0.77, 1.59) | 0.98 (0.71, 1.35) | 0.90 (0.49, 1.66) | 0.77 (0.43, 1.38) |
| **Toilet type** | | | | |
| **Owned pit latrine** | REF | REF | REF | REF |
| **Shared pit latrine** | 1.04 (0.69, 1.57) | 1.07 (0.77, 2.01) | 1.08 (0.58, 2.01) | 1.24 (0.69, 2.23) |
| **Number of children in the house** | 0.99 (0.94, 1.04) | 0.97 (0.93, 1.02) | 1.03 (0.94, 1.12) | 0.77 (0.43, 1.38) |
| **Number of people in the house** | 0.97 (0.93, 1.01) | 0.98 (0.94, 1.01) | 0.96 (0.33, 2.8) | 0.94 (0.88, 1.0) |
